# Conservation seed physiology of the ciénega endemic, *Eryngium sparganophyllum* (Apiaceae)

**DOI:** 10.1093/conphys/coaa017

**Published:** 2020-04-04

**Authors:** Dustin Wolkis, Steve Blackwell, Shyla Kaninaualiʻi Villanueva

**Affiliations:** 1 Department of Science and Conservation, National Tropical Botanical Garden, 3530 Papalina Rd. Kalāheo, HI 96741, USA; 2 Department of Research, Conservation and Collections, Desert Botanical Garden, 1201 N Galvin Pkwy. Phoenix, AZ, 85008, USA

**Keywords:** *Eryngium sparganophyllum*, ciénega, dormancy, morphophysiological, orthodox, conservation seed bank

## Abstract

Knowledge of seed dormancy and optimal propagation techniques is crucial for successful *ex situ* restoration and reintroduction projects, and determining the seed storage behaviour of a species is critical for the long-term conservation of seeds, further supporting future *ex situ* efforts. *Eryngium sparganophyllum* (Apiaceae) is a globally critically endangered plant species endemic to ciénega wetlands of southwest North America. To support *in situ* and *ex situ* conservation efforts of *E. sparganophyllum*, we asked (i) how does the embryo: seed (E:S) ratio change over time once imbibed, (ii) how does germination respond with varying periods of exposure to cold (5°C) and warm (25°C) stratification, and concentrations of gibberellic acid (GA_3_). By answering these questions, (iii) can dormancy class be inferred, and (iv) what storage behaviour category is exhibited? To answer these questions, we collected seeds in Southern Arizona from one of the few remaining wild populations. We measured embryo growth and tested the effects of cold (0–18 weeks) and warm (0 and 4 weeks) stratification, and 0–1000 ppm gibberellic acid on germination. We also tested the effects of cold (−80°C) dry (~20% equilibrium relative humidity) storage on germination. We found that (i) embryos grow inside seeds prior to germination; (ii) compared to control, cold stratification for at least 6 weeks increased germination and warm stratification had no effect; (iii) 1000-ppm GA_3_ had the highest germination success; (iv) therefore this species exhibits morphophysiological dormancy; and (v) seeds are orthodox and can therefore be conserved using conventional storage methods. This information will aid managers in the propagation of *E. sparganophyllum* that is crucial for *in situ* reintroduction and restoration projects, and seed banking represents a critical *ex situ* conservation strategy for the preservation of this species.

## Teaser

Arizona eryngo (*Eryngium sparganophyllum*) is an imperilled plant species. For optimal propagation by seed and to overcome morphophysiological dormancy, *E. sparganophyllum* requires either 6 weeks imbibed at refrigerated conditions (around 5°C) or application of 1000 ppm gibberellic acid. Seeds are orthodox and can be conserved long-term using conventional storage methods.

## Introduction

Common practices involved in managing species of conservation importance include seed collecting, banking and propagation (Guerrant *et al.*, 2004; Convention on Biological Diversity, 2011; Center for Plant Conservation, 2019). Therefore, species specific knowledge of seed storage behaviour and dormancy release are essential for successful *ex situ* species conservation and *in situ* reintroduction and restoration projects. Although dormancy and germination as well as seed storage behaviour in wild species has been studied in detail, many species specific knowledge gaps exist (Walters *et al.*, 2005; Baskin and Baskin, 2014). One such species is *Eryngium sparganophyllum* Hemsl. (Apiaceae), a perennial forb colloquially known as Arizona eryngo (Kearney *et al.*, 1960). *E. sparganophyllum* is a globally critically endangered (NatureServe Web Service, 2019) plant with narrow habitat specificity in ciénegas (freshwater emergent wetlands) restricted to the southwestern USA and northern Mexico. *E. sparganophyllum* populations have been extirpated from two of its six historic locations due to the loss of ciénega habitat owing to groundwater depletion and alterations of waterways (Stromberg *et al.*, in press).

### Dormancy

Reintroduction efforts of native plant species from seed commonly fail often because seed dormancy class and appropriate environmental conditions conducive to optimal germination are unknown (Elzenga *et al.*, 2019). Many species in Apiaceae are reported to exhibit morphological dormancy (MD) or morphophysiological dormancy (MPD). In MD at seed dispersal, the seed coat is water permeable and embryos are small and can be either undifferentiated (i.e. lacking radicle and cotyledon(s); e.g. Orchidaceae), or differentiated (radicle and cotyledon(s) present) but underdeveloped and therefore must grow inside the seed before germination occurs. In MPD, seeds are as described as above in MD but also have a physiological inhibiting mechanism that requires an ecological signal to elicit germination (i.e. physiological dormancy; PD; Baskin and Baskin, 2014). Martin (1946) did not study *Eryngium sparganophyllum* specifically, but describes internal morphology of six species in the genus including *E. aquaticum* and *E. articulatum* and described embryos as linear (i.e. small and several times longer than broad) with the latter species being distinct from others in embryo length. Our initial observations confirm a linear embryo type for *E. sparganophyllum* lending to suspected dormancy classes of MD or MPD. Based on the collection date of six seed accessions listed in the Desert Botanical Garden Living Collections Management System, seed dispersal occurs from August to October (see livingcollections.org). In southern Arizona, the temperatures in the months following this dispersal period are cooler over the winter before warming again in the spring (Western Regional Climate Center, 2019). Therefore, it is possible that this period of cooler temperatures acts as an environmental cue to elicit germination. Other studies have investigated *Eryngium* spp. germination and likely dormancy classes of MD, MPD, or non-dormant (ND) identified. For example, based on Kreiberg (2010), Baskin and Baskin (2014) assume a dormancy class of MPD for the rare facultative salt marsh species *E. armatum*. Some desert annuals in a diversity of families have PD or MPD which is broken by the dry hot season and therefore germinate in the hot wet (i.e. monsoon) and/or in the cool wet season (Baskin and Baskin, 2014). *E. creticum* is such a species where PD was broken by dry ambient lab conditions and incidentally germination was higher in darkness rather than in light (Hammouda and Bakr, 1969) and assumed to have MPD (Baskin and Baskin, 2014). Cold stratification of the shingle beach species *E. maritimum* at 2°C for 6 weeks increased germination 25% at a temperature regime of 25/15°C, but further stratification up to 14 weeks gave variable results and no further benefit (Walmsley and Davy, 1997) and dormancy class is likely MPD (Baskin and Baskin, 2014). In a another study of *E. maritimum*, Necajeva and Ievinsh (2013) find that embryo growth is required prior to germination, and that seeds require cold stratification at 5°C which can be substituted with a treatment of gibberellic acid, confirming a dormancy class of MPD. Greene and Curtis (1950) report *E. yuccifolium* seeds cold stratified for 2 months outdoors during a Wisconsin winter germinated to 40% compared to 0% in unstratified seeds, and Baskin and Baskin (2014) assign a dormancy level and class of deep complex MPD. Elizalde *et al.*, (2007) report that in untreated seeds of *E. horridum* stored at laboratory conditions between 3 and 250 days postharvest, that after 90 days seeds were no longer dormant, suggesting that dry storage at ambient temperature alleviates dormancy. Similarly, for *E. foetidum* seeds stored at ambient conditions for 6 months, the rate of germination increased (but not the final proportion germinated; Fuentes Fiallo *et al.*, 1996), and application of 500 ppm GA_3_ + 50 ppm Kinetin increased germination compared to untreated seeds (Mozumder and Hossain, 2013). Thus, we infer a dormancy class of either MD or MPD for *E. horridum* and *E. foetidum* seeds. Although 45 days of cold stratification accelerated germination in seeds of *E. paniculatum*, Chichizola *et al.* (2019) find no statistical difference between 45 days of cold stratification and control seeds in final germination, concluding that *E. paniculatum* seeds require no germination pretreatment. Thus, here we assign a dormancy class of ND. Stephens *et al.* (2012) report no significant difference in germination in seeds of the rare Floridian *E. cuneifolium* treated with 0–100 ppm GA_3_ at 21–29°C and that no germination occurred at greenhouse temperatures of 18–50°C, so assuming GA_3_ concentrations would have been sufficient to break intermediate complex MPD if present, and although *E. cuneifolium* seeds do not exhibit intermediate complex MPD, no overall dormancy class is here inferred. Sabatino *et al.* (2015) found that there was no difference between mechanically scarified and untreated seeds of *E. regnellii*, so although seeds are not physically dormant (PY) as expected, no dormancy class is here inferred. Of the five species of *Eryngium* reviewed by Baskin and Baskin (2014) and additional five species reviewed above, dormancy class could not be inferred for two species, two species could exhibit either MD or MPD, four species were assumed to exhibit MPD, only *E. petiolatum* was assumed MD, and only *E. paniculatum* was assumed ND.

### Storage behaviour

*Ex situ* seed banking is an important tool for achieving restoration success (e.g. plant/population increase, self-sustaining populations, down-listing species) and provides long-term protection against genotype and/or species loss (Cochrane *et al.*, 2007), and managers are increasingly dependent on seed storage as sources for germplasm (Plant Conservation Alliance, 2015). Thus knowledge of seed storage behaviour and longevity are critical for both *in* and *ex situ* conservation, but managers need to understand *ex situ* survival so that seeds are withdrawn before a decline to an unacceptable level is reached (Guerrant and Fielder, 2004; Plant Conservation Alliance, 2015). Guerrant and Raven (1998) found that 100% of unreplicated *E. petiolatum* seeds germinated after desiccation to 15% RH and frozen, indicating seeds are likely orthodox. However, germination decreased slightly but significantly in *E. horridum* seeds stored at ambient laboratory conditions after 250 days (Elizalde *et al.*, 2007). Similarly, *Eryngium foetidum* seeds lost viability at ambient storage conditions after nine months (Fuentes Fiallo *et al.*, 1996). In temperate montane grasslands in central Argentina, *E*. *agavifolium* and *E. nudicaule* persisted in the soil seed bank for less than 1 year (Funes *et al.*, 1999), and the Seed Information Database (SID; Royal Botanic Gardens Kew, 2019) reports a storage behaviour of ‘uncertain’ for both species. For the remaining 26 species of *Eryngium* assessed in SID, all are reported as either orthodox or probably orthodox (Royal Botanic Gardens Kew, 2019).

With respect to *E. sparganophyllum* we ask (i) how does the embryo:seed (E:S) ratio change over time once imbibed; how does germination respond after varying (ii) lengths of time in cold (5°C) and warm (25°C) stratification and (iii) concentrations of gibberellic acid (GA_3_); by synthesizing answers to the above, (iv) can dormancy class be inferred, and (v) how can dormancy be alleviated with highest germination success; and to preserve seeds for future reintroduction and restoration projects (vi) what storage behaviour category is exhibited? By answering these questions, we hope to provide vital information on the conservation seed physiology of this globally critically endangered species to aid conservation and restoration practitioners with information on germination, optimal seed propagation techniques and seed storage behaviour and longevity.

## Materials and methods

### Field

All *Eryngium sparganophyllum* seeds were collected from La Cebadilla Ciénega east of Tucson, Arizona, USA, above Tanque Verde Wash in the Arizona Upland Subdivision—Sonoran Desert Scrub (Brown *et al.*, 2007). Herbarium voucher specimens for this population are on file at herbaria ASU and DES (see collector and collector number Liz Makings 4459 and Dustin Wolkis 452). Seeds used in the temperature and GA_3_ experiments (DBG-2016-0009-10) were collected from ~ 50 individual plants on 25 January 2016 and kept in paper coin envelopes at ambient laboratory conditions (~25°C/20%RH) until experiments commenced on 31 December 2014. Seeds used to measure embryo growth (DBG-2016-0010-10/NTBG-20170652) were collected from ~ 50 individual plants on 17 September 2015, and seeds used to study storage behaviour (DBG-2016-0184-10/NTBG-20170653) were collected from ~ 100 individual plants on 29 August 2016. After collection, seeds used in both the embryo growth and storage behaviour studies were stored at ambient laboratory conditions (~25°C/20% RH) until they were tested for viability on 26 October 2017, then desiccated to ~ 42% eRH at 20°C, then hermetically sealed and placed in storage at −80°C (achieving a target eRH of ~20%) until used in experiments.

### Embryo growth

One hundred *E. sparganophyllum* seeds were withdrawn from storage and imbibed overnight in a solution of 1000 ppm GA_3_ and distilled water. To determine the initial embryo:endosperm (E:S) ratio, the next day embryos from 15 seeds were excised and embryo and endosperm length measured under a dissecting microscope. The remaining seeds were sown in a 60-mm Petri dish on blotter paper moistened with a solution of 0.1% solution of a plant preservative mixture (PPM; Plant Cell Technology) in distilled water to inhibit fungal growth without affecting germination (Assaf Guri, personal communication), sealed with plastic paraffin film to increase water retention and placed in a germination chamber (Percival GR36L) with a 12/12-h photoperiod and a daily alternating temperature regime of 25/15°C. Seeds in the petri dish were monitored weekly until the first signs of radicle emergence. To determine the critical E:S ratio, 15 seeds whose radicles have started to penetrate the seed coat but have not yet emerged were dissected and final embryo and endosperm lengths measured under a dissecting microscope. The E:S ratio is defined as embryo length divided by endosperm length.

### Temperature

To determine the effect of cold and warm stratification on germination, three replicates (see, Baskin and Baskin, 2014) of 16 seeds each were sown on sterilized silica sand moistened with room temperature (~25°C) distilled water. Because it was not possible to control photoperiod during stratification (and because the effects of such are unknown for this species), seeds were placed in the dark. To determine the effect of cold stratification, we placed seeds at 5°C from 0 (control) to 18 weeks in 2-week intervals. To determine the effect of warm stratification, we used the control from the above and also placed seeds at 25°C for 2 weeks.

### Gibberellic acid

To determine the germination response to gibberellic acid (GA_3_), three replicates of 16 seeds each were soaked in solutions of 0 (control) 250, 500, 750 and 1000 ppm GA_3_ and distilled water at ambient laboratory temperature (~25°C) for 24 h and not rinsed before sown.

### Storage behaviour and longevity

After collection, seeds were stored at ambient laboratory conditions (~25°C and 20% RH) in open containers for 423 days until viability was assessed. To achieve a storage eRH of ~20%, the entire seed accession was placed in a humidity chamber at ~ 42% RH at passive temperature (~20°C) for 30 days (Walters, 2004). The accession was then hermetically sealed in tri-laminate aluminium foil pouches, and frozen to −80°C for 256 days until viability was again assessed.

### Germination

For the temperature and GA_3_ experiments seeds were sown in 90-mm Petri dishes on silica sand moistened with sterilized distilled water. Each Petri dish was placed inside a resalable zip-top plastic bag to preserve moisture. Each replicate was placed in a Hoffman germination chamber at a daily alternating 12/12-h photoperiod and 20/15°C thermocycle. Seeds were assayed at 2–3-day intervals until 30 days had elapsed since entering the germination chamber. Germinated seeds, indicated by radicle emergence, were removed from each test at the time of observation and grown at Desert Botanical Garden. Seeds that did not germinate at the conclusion of each test were not further examined.

For the storage behaviour study, three replicates of 20 (pre-dry-cold-storage) and 40 (post-dry-cold-storage) seeds were sown in 60-mm Petri dishes on blotter paper moistened with a solution of 0.1% plant preservative mixture (PPM; Plant Cell Technology, Inc) in distilled water to inhibit fungal growth without affecting germination (Assaf Guri, personal communication), sealed with plastic paraffin film to increase water retention and placed in a germination chamber (Percival GR36L) at a daily alternating 12 h light (~50 μmol m^−2^ second^−1^ cool white (4100 K) fluorescent light)/12-h dark photoperiod and 25/15°C temperature regime. Each replicate was monitored every two (occasionally three) weeks until either every seed had germinated (indicated by radicle emergence) or died (attacked by fungus) or until 1 year had elapsed since date sown. Non-germinated seeds were not further investigated, and germinated seeds were immediately discarded.

**Figure 1:**
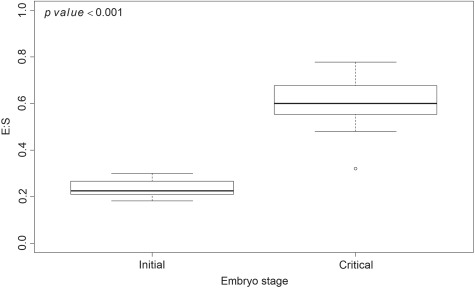
Box plots showing the initial and critical E:S (embryo to seed ratio). Lower line is minimum, lower end of box is the first quartile, bold horizontal line is median, upper end of box is the third quartile, and upper line is maximum, and the open circle is an outlier.

**Figure 2:**
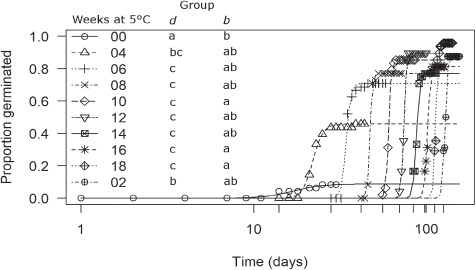
Germination time courses for lengths of time cold stratified (5°C). Groups with the same letter indicate that there is no significant difference (*α* = 0.05) in parameter estimates *d* (maximum germination), and absolute value *b* (proportional to slope). Time to 50% germination (t_50_) was not biologically meaningful since t_0_ differed for each length of time in cold stratification and has therefore been omitted. Unlabeled tick marks along the x-axis indicate when seeds were moved into germination conditions.

### Statistical analysis

Germination data was analyzed using a time-to-event model (Ritz *et al.*, 2013) with the ‘drc’ package (Ritz *et al.*, 2015) for the software environment R (R Core Team, 2018) and RStudio (RStudio Team, 2016). A non-linear log-logistic three-parameter model was used:

$$ F(t)=\frac{d}{1+\exp \left[b\log (t)-\log \left({t}_{50}\right)\right]} $$

where parameter estimates: *d* is maximum germination; *t*_50_ is the time where 50% of the seeds germinated; and *b* is proportional to the slope of *F* at time *t*. For one replicate in the GA_3_ experiment (750 ppm), and for two replicates in the cold stratification experiment (4 and 16 weeks, respectively), *d* was substituted for the actual proportion germinated. For GA_3_ and cold stratification experiments the three parameter estimates (or actuals in the cases of the above) were compared using analysis of variance and post hoc Tukey tests, and for the warm stratification experiment a *t* test was used with the ‘CompParm’ (Ritz *et al.*, 2015) function (*α* = 0.05). The corrected *z*-test proposed by Ellis *et al*. (1985) was used to determine if a decline in viability had occurred in accession number DBG-2016-0184-10 (NTBG-20170653). Means are reported with standard deviation.

**Table 1:** Length of time in cold (5°C) stratification pairwise *P* value comparisons obtained from post hoc Tukey test for parameter estimates *d* (maximum germination), and absolute value *b* (proportional to slope)

**Weeks at 5 °C**	***P* value**	
** Comparison**	***d***	***b***
02–00	0.010	0.998
04–00	<0.001	0.995
06–00	<0.001	0.093
08–00	<0.001	0.169
10–00	<0.001	0.022
12–00	<0.001	0.387
14–00	<0.001	0.073
16–00	<0.001	0.031
18–00	<0.001	0.011
04–02	0.163	1.000
06–02	0.040	0.358
08–02	<0.001	0.541
10–02	<0.001	0.106
12–02	0.040	0.842
14–02	0.014	0.298
16–02	<0.001	0.150
18–02	<0.001	0.055
06–04	0.999	0.413
08–04	0.780	0.605
10–04	0.491	0.130
12–04	0.999	0.886
14–04	0.960	0.348
16–04	0.162	0.181
18–04	0.638	0.068
08–06	0.991	1.000
10–06	0.892	0.999
12–06	1.000	0.996
14–06	1.000	1.000
16–06	0.490	1.000
18–06	0.960	0.983
10–08	1.000	0.985
12–08	0.991	1.000
14–08	1.000	1.000
16–08	0.960	0.996
18–08	1.000	0.918
12–10	0.892	0.846
14–10	0.991	1.000
16–10	0.999	1.000
18–10	1.000	1.000
14–12	1.000	0.991
16–12	0.490	0.918
18–12	0.960	0.661
16–14	0.780	1.000
18–14	0.999	0.993
18–16	0.991	1.000

Time to 50% germination (*t*_50_) was not biologically meaningful since *t*_0_ differed for each length of time in cold stratification and has therefore been omitted.

## Results

### Embryo growth

In accession NTBG-20170652 (DBG-2016-0010-10) mean initial and critical embryo lengths were 0.55 mm (0.76 SD), and 1.51 mm (3.37 SD), respectively, indicating a 155% increase in 15 days. Mean initial embryo:seed (E:S) ratio was 0.2363 (0.0337 SD) and mean critical E:S ratio was 0.6023 (0.1153 SD), with a significant difference between the initial and critical E:S ratios (*P* < 0.001; Fig. 1).

### Temperature

**Figure 3:**
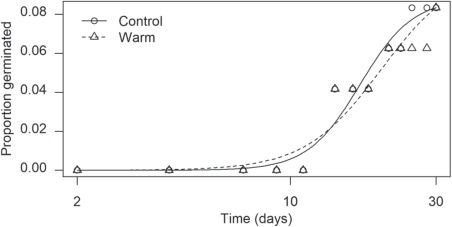
Germination time courses for warm (25°C) stratification for 0 (control) and 2 weeks. There were no statistical differences for any parameter estimates (all *P* values > 0.711)

For length of time in cold stratification, mean parameter estimates ranged for maximum proportion germinated (*d*) from 0.09 (control; 0.08 SD) to 0.97 (16 weeks; 0.04 SD) and for absolute value of *b* from 3.62 (control; 3.54 SD) to 77.44 (18 weeks; 35.41 SD). Time to 50% germination (*t*_50_) was not biologically meaningful since *t*_0_ differed for each length of time in cold stratification. Statistically significant differences in *d* were detected between control (0 weeks) and all lengths of time cold stratified (all *P* values < 0.011): 2 weeks and 6 through 18 weeks cold scarified (all *P* values < 0.0399). Statistical differences for absolute value of *b* were between control and 10, 16 and 18 weeks cold stratified (all *P* values < 0.031; Fig. 2; Table 1). There was no difference between warm stratification and control for *d* (0.10 (0.07 SD) and 0.09 (0.04 SD), respectively; *P* = 0.837), *t*_50_ (19.78 (9.24 SD) and 16.91 (3.39 SD); *P* = 0.739), or absolute value of *b* (3.49 (2.33 SD) and 5.05 (2.56), respectively; *P* = 0.712; Fig. 3). Seeds started germinating during cold stratification starting at Week 8 (0.02%), and then consecutively at Weeks 10 (0.04%), 12 (17%), 14 (17%), 16 (29%) and 18 (29%).

**Figure 4:**
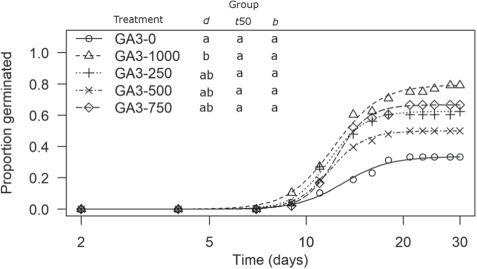
Germination time courses for varying concentrations of gibberellic acid (GA3). Groups with the same letter indicate that there is no significant difference (*α* = 0.05) in parameter estimates *d* (maximum germination), t50 (time to 50% germination), and absolute value *b* (proportional to slope).

### Gibberellic acid

In the gibberellic acid (GA_3_) experiments mean parameter estimates ranged for maximum proportion germinated (*d*) from 0.33 (control; 0.09 SD) to 0.80 (1000 ppm GA_3_; 0.14 SD); time to 50% germination (*t*_50_) from 11.90 days (500 ppm GA_3_; 1.08 SD) to 14.73 days (750 ppm GA_3_; 2.78 SD); and for absolute value of *b* from 6.03 (750 ppm GA_3_; 3.39 SD) to 8.71 (250 ppm GA_3_; 1.86 SD). The only statistically significant difference in *d* was between control (0 ppm GA_3_) and 1000 ppm GA_3_ (*P* = 0.013) with all other concentrations of GA_3_ exhibiting an intermediate response. There were no statistical differences for *t*_50_ or *b* (Fig. 4; Table 2).

### Storage behaviour

Proportion germinated after 423 days at ambient laboratory conditions was 0.42 (0.13 SD), and after subsequent desiccation and freezing at −80°C for 256 days was 0.28 (0.04 SD). A viability decline was detected in this accession (*z* = 1.83).

## Discussion

### Dormancy

There was a significant difference between initial and critical E:S ratios (Fig. 1) indicating that embryos grew inside the seeds prior to radicle emergence, and thus seeds of this species exhibit morphological (MD) or morphophysiological (MPD) dormancy. Cold stratification at 5°C for at least 6 weeks increased maximum germination compared to control (*d*; Fig. 2; Table 1), while warm stratification had no effect on any parameter estimates (Fig. 3). Addition of GA_3_ at 1000 ppm increased maximum germination (*d*) but had no effect on the other parameter estimates (Fig. 3; Table 2). Thus, seeds of *E. sparganophyllum* exhibit MPD. Further, some seeds did germinate (thus embryos grew) at 5°C during the cold stratification, yet the majority of germination (radicle emergence) occurred after cold stratification, and GA_3_ also promoted germination (possibly substituting for cold stratification), thus a level of intermediate complex MPD can be inferred (see, Baskin and Baskin, 2014). Although *E. paniculatum* is assumed to be non-dormant (based on Chichizola *et al.*, 2019), and *E. petiolatum* was assumed to exhibit MD (Baskin and Baskin, 2014), other *Eryngium* species including *E. armatum*, *E. creticum*, *E. maritimum* and *E. yuccifolium* are also assumed to exhibit MPD (Necajeva and Ievinsh, 2013; Baskin and Baskin, 2014). Although the effects of an alternating light/dark photoperiod versus constant darkness was not tested in this investigation, seed germination in *E. caeruleum* and *E. ovinum* were both inhibited by constant darkness (Morgan, 1998; Rezvani and Zaefarian, 2017). However, Hammouda and Bakr (1969) found higher germination in dark vs light for *E. creticum* seeds. More research is needed to test the effects of light/dark on the dormancy and germination of *E. sparganophyllum*.

**Table 2:** Gibberellic acid (GA3) pairwise *P* value comparisons obtained from post hoc Tukey test for parameter estimates *d* (maximum germination), *t*_50_ (time to 50% germination) and absolute value *b* (proportional to slope)

**Treatment comparison**	***P* value**		
	***d***	***t***_**50**_	***b***
GA_3_-1000—GA_3_-0	0.013	0.990	0.991
GA_3_-250—GA_3_-0	0.139	0.972	0.832
GA3-500—GA3-0	0.591	0.947	0.858
GA_3_-750—GA_3_-0	0.078	0.690	0.995
GA_3_-250—GA_3_-1000	0.555	1.000	0.972
GA_3_-500—GA_3_-1000	0.126	0.999	0.980
GA_3_-750—GA_3_-1000	0.765	0.438	0.921
GA_3_-500—GA_3_-250	0.786	1.000	1.000
GA_3_-750—GA_3_-250	0.995	0.370	0.634
GA_3_-750—GA_3_-500	0.578	0.317	0.666

### Storage behaviour

Although a viability decline was detected in the one accession tested, it should be noted that no dormancy breaking pre-treatments were used, and that the first test was monitored for 1 year, while the second test was terminated after only 80 days because non-germinated seeds became parasitized by a black fungus. In the first test, 15% of seeds germinated after 80 days and the last germination event occurred between 334 and 348 days. It is possible that given equal monitoring periods the post desiccation/freezing seeds would have germinated to a similar proportion. Seeds survived desiccation to around 20% eRH and subfreezing temperatures of −80°C; therefore, *E. sparganophyllum* is likely orthodox and may be preserved using conventional storage methods (or at −80°C) for periods of decades to centuries (Walters *et al.*, 2005). This result agrees with other species of *Eryngium*. For example, 100% of unreplicated *E. petiolatum* seeds germinated after desiccation to 15% RH and frozen (Guerrant and Raven, 1998). However, storage of *E. maritimum* seeds desiccated to unknown levels at a mean temperature of 2°C for 7 years did not affect innate dormancy, yet germination was reduced and *t*_50_ increased (Walmsley and Davy, 1997). Germination at ambient storage conditions decreased after 3 months in *E. horridum* (Elizalde *et al.*, 2007) and after 9 months in *E. foetidum* (Fuentes Fiallo *et al.*, 1996). It is possible that the 14 months exposed to good although less than ideal ambient conditions (25°C/20% RH) before storage in optimal *ex situ* conditions decreased long-term viability (see Probert, 2003).

### Disturbance

Within a soil seed bank, seeds may be able to survive fire (Overbeck *et al.*, 2006) but fire also has the potential to kill seeds or to promote germination (Walck *et al.*, 2011). There is evidence that ciénegas were burned probably as a management tool prior to contact with introduced species (Davis *et al.*, 2002), and recent fire within ciénegas is evident (Wolkis, 2016), suggesting that ciénegas may be resilient to burning. *E. horridum* and *E. pristis* seeds died after a 2-minute exposure to 150 and 180°C, respectively, but survived after 2 minutes exposed to 130°C (27 and 8%, respectively; Overbeck *et al.*, 2006). Thus, it is possible that *E. sparganophyllum* seeds could also survive high temperatures associated with fire. More research is needed to fully understand the survival of seeds of this species at high temperatures. Insects are the most import group of pre-dispersal seed predators and can consume the entire reproductive output of host plants (Schowalter, 2016). Thus, pre-dispersal seed predation by insects can be a major challenge to rare plant conservation (Ancheta and Heard, 2011). Microlepidopteran larvae (Gelechiidae) have been reported to burrow into the centre of the inflorescence of *E. yuccifolium* predating and destroying 40–60% of ovaries and seeds (Molano-Flores, 2001). A wide taxonomic invertebrate diversity has been documented on and in *E. sparganophyllum* including Bombyliidae (bee fly), Buprestid beetle, *Crematogaster* sp. (acrobat ant), *Campsomeris tolteca* (scoliid wasp), *Gasteruption* sp. (carrow wasp), *Jalysus* sp. (stilt bug), *Myzinum quinquecinctum* (five-banded tiphiid wasp), Oxyopidae (lynx spider), *Phaneroptera* sp. (katydid nymph), *Poecilognathus* sp. (bee fly), *Polistes* sp. (paper wasp), *Spragueia magnifica* (Noctuid moth) and *Zelus* sp. (Assassin bug; Stromberg *et al.*, in press). More research is needed to elucidate the effects of these plant–animal interactions on seed viability.

## Conclusions

The globally critically imperilled ciénega endemic, *Eryngium sparganophyllum* (NatureServe Web Service, 2019), has been extirpated from of two of its six historic locations (Stromberg *et al.*, in press), and restoration and reintroduction projects are currently underway. Thus, it is crucial for managers to successfully propagate this species, especially by seed. Seeds of *E. sparganophyllum* exhibit intermediate complex MPD, and dormancy breaking pre-treatments of 6 weeks of cold stratification at 5°C or 1000 ppm GA_3_ are sufficient to stimulate germination. Seeds of this species are orthodox and can be stored long-term by conventional storage methods. Therefore, seed banking represents a critical *ex situ* conservation tool for the preservation of this species.

## References

[ref1] Ancheta J, Heard SB (2011) Impacts of insect herbivores on rare plant populations. Biol Conserv 144: 2395–2402.

[ref2] Baskin CC, Baskin JM (2014) Seeds: Ecology, Biogeography and Evolution of Dormancy and Germination, EdEd 2 Academic Press, San Diego.

[ref3] Brown DE, Brennan TC, Unmack PJ (2007) A digitized biotic community map for plotting and comparing North American plant and animal distributions. CANOTIA 3: 1–12.

[ref4] Center for Plant Conservation (2019) CPC Best Plant Conservation Practices to Support Species Survival in the Wild In Escondido

[ref5] Chichizola GA, Rovere AE, Gonzalez SL (2019) Germinación de Phacelia secunda (Boraginaceae) y Eryngium paniculatum (Apiaceae), hierbas perennes de la Patagonia Argentina. Rev Peru Biol 26 26: 311–316.

[ref6] Cochrane JA, Crawford AD, Monks LT (2007) The significance of ex situ seed conservation to reintroduction of threatened plants. Aust J Bot 55: 356–361.

[ref7] Convention on Biological Diversity (2011) Global Strategy for Plant Conservation https://www.cbd.int/gspc/

[ref8] Davis OK, Minckley TA, Moutoux T, Jull T, Kalin B (2002) The transformation of Sonoran Desert wetlands following the historic decrease of burning. J Arid Environ 50: 393–412.

[ref9] Elizalde JHI, García LF, Maidana AC, Lallana VH (2007) Germinación y viabilidad de semillas de Eryngium horridum malme almacenadas en laboratorio. Rev Científica Agropecu 11: 121–127.

[ref10] Ellis RH, Hong TD, Roberts EH (1985) Handbook of Seed Technology for Genebanks. International Plant Genetic Resources Institute (IPGRI), Rome.

[ref11] Elzenga JTM, Bekker RM, Pritchard HW (2019) Maximising the use of native seeds in restoration projects. Plant Biol 21: 377–379. 30977290 10.1111/plb.12984 PMC6594131

[ref12] Fuentes Fiallo VR, Rodríguez Medina NN, Rodríguez Ferradá CA (1996) La germinacion del culantro (Eryngium foetidum L.). Rev Cuba Plantas Med 1: 31–33.

[ref13] Funes G, Basconcelo S, Díaz S, Cabido M (1999) Seed size and shape are good predictors of seed persistence in soil in temperate mountain grasslands of Argentina. Seed Sci Res 9: 341–345.

[ref14] Greene H, Curtis J (1950) Germination studies of Wisconsin prairie plants. Am Midl Nat 43: 186–194.

[ref15] Guerrant EO, Fielder PL (2004) Accounting for sample decline during ex situ storage and reintroduction In Guerrant EO, Havens K, Maunder M, eds, Ex Situ Plant Conservation: Supporting Species Survival in the Wild. Island Press, Washington, DC, pp. 365–386.

[ref16] Guerrant EO, Havens K, Maunder M (eds) (2004) Ex Situ Plant Conservation Supporting Species Survival in the Wild. Island Press, Washington.

[ref17] Guerrant EO, Raven A (1998) Seed germination and storability studies of 69 plant taxa native to the Willamette valley Wet prairie In Rose R, Haase DL, eds, Symposium Proceedings Native Plants Propagating and Planting. Oregon State University, College of Forestry, Nursery Technology Cooperative, Corvallis, pp. 25–31.

[ref18] Hammouda M, Bakr Z (1969) Some aspects of germination of desert seeds. Phyt 13: 183–201.

[ref19] Kearney T, Peebles J, Howell J, McClintock E (1960) Arizona Flora, Revised, EdEd 2 University of California Press, Berkeley.

[ref20] Kreiberg P (2010) Can Seed Treatments Improve Germination of Rare Salt Marsh Species? Combined Proceedings of the International Plant Propagators Society, In, pp. 583–584.

[ref21] Martin AC (1946) The comparative internal morphology of seeds. Am Midl Nat 36: 513–660.

[ref22] Molano-Flores B (2001) Reproductive biology of Eryngium yuccifolium (Apiaceae), a prairie species. J Torrey Bot Soc 128: 1–6.

[ref23] Morgan JW (1998) Comparative germination responses of 28 temperate grassland species. Aust J Bot 46: 209–219.

[ref24] Mozumder SN, Hossain MM (2013) Effect of seed treatment and soaking duration on germination of Eryngium foetidum L. seeds. Int J Hortic 3: 46–51.

[ref25] NatureServe Web Service (2019) Natureserve. http://services.natureserve.org (last accessed 6 May 2019).

[ref26] Necajeva J, Ievinsh G (2013) Seed dormancy and germination of an endangered coastal plant Eryngium maritimum (Apiaceae). Est J Ecol 62: 150.

[ref27] Overbeck GE, Müller SC, Pillar VD, Pfadenhauer J (2006) No heat-stimulated germination found in herbaceous species from burned subtropical grassland. Plant Ecol 184: 237–243.

[ref28] Plant Conservation Alliance (2015) National Seed Strategy for Rehabilitation and Restoration.

[ref29] Probert RJ (2003) Seed viability under ambient conditions, and the importance of drying In Smith RD, Dickie JB, Linnington SH, Pritchard HW, Probert RJ, eds, Seed Conservation: Turning Science into Practice. The Royal Botanic Gardens, Kew, Richmond, p. 1023.

[ref30] R Core Team (2018) R: A language and environment for statistical computing.

[ref31] Rezvani M, Zaefarian F (2017) Effect of some environmental factors on seed germination of Eryngium caeruleum M. Bieb. populations. Acta Bot Brasilica 31: 220–228.

[ref32] Ritz C, Baty F, Streibig JC, Gerhard D (2015) Dose-response analysis using R. PLoS One 10: 1–13. 10.1371/journal.pone.0146021 PMC4696819 26717316

[ref33] Ritz C, Pipper CB, Streibig JC (2013) Analysis of germination data from agricultural experiments. Eur J Agron 45: 1–6.

[ref34] Royal Botanic Gardens Kew (2019) Seed Information Database (SID). Version 7.1 http://data.kew.org/sid

[ref35] RStudio Team (2016) RStudio: Integrated Development for R. In

[ref36] Sabatino M, Rovere A, Maceira N (2015) Germinación de Eryngium regnellii: especie clave para la restauración ecológica de las interacciones planta-polinizador en la Pampa Austral (Buenos Aires, Argentina). Rev Int Botánica Exp 9457: 435–443.

[ref37] Schowalter TD (2016) Pollination, seed predation, and seed dispersal In Insect Ecology, EdFourth Academic Press, pp. 445–476.

[ref38] Stephens EL, Castro-Morale L, Quintana-Ascencio PF (2012) Post-dispersal seed predation, germination, and seedling survival of five rare Florida scrub species in intact and degraded habitats. Am Midl Nat 167: 223–239.

[ref39] Stromberg JC, Makings E, Wolkis D, Brown DE (in press) Conservation of the Arizona Ciénega Endemic. Eryngium sparganophyllum Hemsl, Southwest Nat.

[ref40] Walck J, Siti H, Dixon KW, Ken T, Poschlod P (2011) Climate change and plant regeneration from seed. Glob Chang Biol 17: 2145–2161.

[ref41] Walmsley C, Davy A (1997) Germination characteristics of shingle beach species, effects of seed ageing and their implications for vegetation. J Appl Ecol 34: 131–142.

[ref42] Walters C (2004) Principles for preserving germplasm in gene banks In Guerrant EO, Havens K, Maunder M, eds, Ex Situ Plant Conservation: Supporting Species Survival in the Wild. Island Press, Washington D.C., pp. 113–138.

[ref43] Walters C, Wheeler LM, Grotenhuis JM (2005) Longevity of seeds stored in a genebank: species characteristics. Seed Sci Res 15: 1–20.

[ref44] Western Regional Climate Center (2019) Cooperative Climatological Data Summaries. https://wrcc.dri.edu/

[ref45] Wolkis D (2016) Plant Ecology of Arid land Wetlands; A Watershed Moment for Plant Conservation. Arizona State University, MS Thesis.

